# 5-Bromo-2,3-dihydro-1*H*-cyclo­penta­[*a*]naphthalen-1-one

**DOI:** 10.1107/S1600536809031547

**Published:** 2009-08-19

**Authors:** Liang Zhang, Di Sun, Xiang-Zhi Gao, Su-Yuan Xie, Rong-Bin Huang

**Affiliations:** a Department of Chemistry, College of Chemistry and Chemical Engineering, Xiamen University, Xiamen 361005, People’s Republic of China; b State Key Laboratory for Physical Chemistry of Solid Surfaces, Xiamen University, Xiamen 361005, People’s Republic of China

## Abstract

The title compound, C_13_H_9_BrO, has been synthesized by the intra­molecular Friedel–Crafts reaction of 1-(1-bromo-4-naphth­yl)-3-chloro­propan-1-one. There are two approximately planar [maximum deviations of 0.8 (2) and 0.4 (2) Å in the two mol­ecules] molecules in the asymmetric unit. The dihedral angle between their mean planes is 19.72 (8)°. Weak inter­molecular C—H⋯O hydrogen bonding is present in the crystal structure.

## Related literature

The trimer of the title compound is a potential inter­mediate in the synthesis of fullerenes, see: Boorum *et al.* (2001 ▶); Scott *et al.* (1996 ▶). The Aldol cyclo­trimerization of the title compound is widely used in the synthesis of fullerenes and bowl-shaped compounds, see: Amick & Scott (2007 ▶). For a related structure, see: Sil *et al.* (2004 ▶).
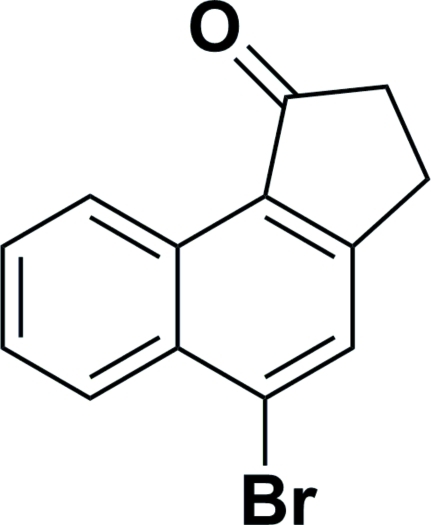


## Experimental

### 

#### Crystal data

C_13_H_9_BrO
*M* *_r_* = 261.10
Triclinic, 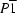

*a* = 7.369 (2) Å
*b* = 9.986 (3) Å
*c* = 14.177 (5) Å
α = 87.763 (6)°
β = 78.991 (6)°
γ = 87.154 (7)°
*V* = 1022.3 (5) Å^3^
*Z* = 4
Mo *K*α radiation
μ = 3.99 mm^−1^
*T* = 298 K
0.09 × 0.08 × 0.06 mm

#### Data collection

Oxford Gemini S Ultra diffractometer
Absorption correction: multi-scan (*CrysAlis RED*; Oxford Diffraction, 2008 ▶) *T*_min_ = 0.716, *T*_max_ = 0.796
5159 measured reflections
3524 independent reflections
2663 reflections with *I* > 2σ(*I*)
*R*_int_ = 0.023

#### Refinement

*R*[*F* ^2^ > 2σ(*F* ^2^)] = 0.041
*wR*(*F* ^2^) = 0.119
*S* = 0.97
3524 reflections
271 parameters
H-atom parameters constrained
Δρ_max_ = 0.52 e Å^−3^
Δρ_min_ = −0.40 e Å^−3^

### 

Data collection: *CrysAlis CCD* (Oxford Diffraction, 2008 ▶); cell refinement: *CrysAlis RED* (Oxford Diffraction, 2008 ▶); data reduction: *CrysAlis RED*; program(s) used to solve structure: *SHELXS97* (Sheldrick, 2008 ▶); program(s) used to refine structure: *SHELXL97* (Sheldrick, 2008 ▶); molecular graphics: *SHELXL97*; software used to prepare material for publication: *SHELXL97* and *publCIF* (Westrip, 2009 ▶).

## Supplementary Material

Crystal structure: contains datablocks I, global. DOI: 10.1107/S1600536809031547/xu2577sup1.cif

Structure factors: contains datablocks I. DOI: 10.1107/S1600536809031547/xu2577Isup2.hkl

Additional supplementary materials:  crystallographic information; 3D view; checkCIF report

## Figures and Tables

**Table 1:** Hydrogen-bond geometry (Å, °)

*D*—H⋯*A*	*D*—H	H⋯*A*	*D*⋯*A*	*D*—H⋯*A*
C15—H15*A*⋯O1^i^	0.97	2.54	3.495 (5)	167

Symmetry code: (i) 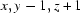.

**Fig. 1.**
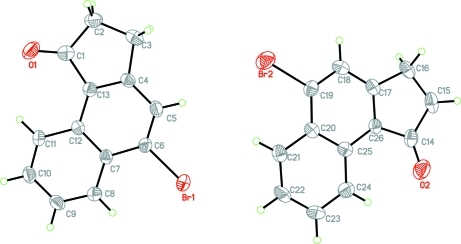
The molecular structure of the title compound with 30% probability displacement ellipsoids.

## supplementary crystallographic information

### Comment

The title compound is the medial compound for the synthesis of its trimmer molecule. In the acid conditions, the trimmer molecule can be obtained by the Aldol cyclotrimerization of 5-bromo-2,3-dihydrocyclopenta[a]naphthalen-1-one. These kinds of the trimmers are the potential intermediate in the synthesis of fullerenes (Boorum *et al.*, 2001; Scott *et al.*, 1996). So this method was widely used in the organic synthesis of fullerenes and bowl-shaped compounds (Amick & Scott, 2007).

The molecular structure is depicted in Fig. 1. Bond lengths and angles are in good agreement with previous reported for similar compounds (Sil *et al.*, 2004). The molecule assumes a co-planar structure, except methylene H atoms. The asymmetric unit of the crystal structure contains two independent molecules, the two molecular planes make a dihedral angle of 19.72 (8)° with respect to each other. Weak intermolecular C—H···O hydrogen bonding (Table 1) is present in the crystal structure(Fig. 2).

### Experimental

All reagents and solvents were used as obtained commercially without further purification. The title compound was synthesized by adding 1-(1-bromonaphthalen-4-yl)-3-chloropropan-1-one (1.4 mL) to concentrated H_2_SO_4_ (11 ml) at room temperature. The resulting mixture was stirred at 383 K for 3 h, after cooling to room temperature, the mixture was poured into water-ice slowly. The aqueous layer was extracted with cyclohexane (3 × 60 mL). The organic layers were combined and washed with saturated NaHCO_3_ solution (120 mL), saturated brine (3 × 60 ml), and dried over MgSO_4_, and concentrated under reduced pressure to provide the title compound. The compound was dissolved in CH_2_Cl_2_ and kept in darkness for several days, yellow block-shaped single crystals were obtained.

### Refinement

H atoms were generated geometrically with C—H 0.93 or 0.97 Å and were allowed to ride on their parent atoms in the riding model approximations, with U_iso_(H) = 1.2U_eq_(C).

### Crystal data

**Table:**

C_13_H_9_BrO	*Z* = 4
*M**_r_* = 261.10	*F*(000) = 520
Triclinic, *P*1	*D*_x_ = 1.696 Mg m^−^^3^
Hall symbol: -P 1	Mo *K*α radiation, λ = 0.71073 Å
*a* = 7.369 (2) Å	Cell parameters from 2244 reflections
*b* = 9.986 (3) Å	θ = 5.0–51.6°
*c* = 14.177 (5) Å	µ = 3.99 mm^−^^1^
α = 87.763 (6)°	*T* = 298 K
β = 78.991 (6)°	Block, yellow
γ = 87.154 (7)°	0.09 × 0.08 × 0.06 mm
*V* = 1022.3 (5) Å^3^	

### Data collection

**Table:**

Oxford Gemini S Ultra diffractometer	3524 independent reflections
Radiation source: fine-focus sealed tube	2663 reflections with *I* > 2σ(*I*)
graphite	*R*_int_ = 0.023
Detector resolution: 16.1903 pixels mm^-1^	θ_max_ = 25.0°, θ_min_ = 2.0°
ω scans	*h* = −8→8
Absorption correction: multi-scan (*CrysAlis RED*; Oxford Diffraction, 2008)	*k* = −11→7
*T*_min_ = 0.716, *T*_max_ = 0.796	*l* = −16→16
5159 measured reflections	

### Refinement

**Table:**

Refinement on *F*^2^	Primary atom site location: structure-invariant direct methods
Least-squares matrix: full	Secondary atom site location: difference Fourier map
*R*[*F*^2^ > 2σ(*F*^2^)] = 0.041	Hydrogen site location: inferred from neighbouring sites
*wR*(*F*^2^) = 0.119	H-atom parameters constrained
*S* = 0.97	*w* = 1/[σ^2^(*F*_o_^2^) + (0.0728*P*)^2^] where *P* = (*F*_o_^2^ + 2*F*_c_^2^)/3
3524 reflections	(Δ/σ)_max_ < 0.001
271 parameters	Δρ_max_ = 0.52 e Å^−^^3^
0 restraints	Δρ_min_ = −0.40 e Å^−^^3^

### Special details

**Table:**

Geometry. All e.s.d.'s (except the e.s.d. in the dihedral angle between two l.s. planes) are estimated using the full covariance matrix. The cell e.s.d.'s are taken into account individually in the estimation of e.s.d.'s in distances, angles and torsion angles; correlations between e.s.d.'s in cell parameters are only used when they are defined by crystal symmetry. An approximate (isotropic) treatment of cell e.s.d.'s is used for estimating e.s.d.'s involving l.s. planes.
Refinement. Refinement of *F*^2^ against ALL reflections. The weighted *R*-factor *wR* and goodness of fit *S* are based on *F*^2^, conventional *R*-factors *R* are based on *F*, with *F* set to zero for negative *F*^2^. The threshold expression of *F*^2^ > σ(*F*^2^) is used only for calculating *R*-factors(gt) *etc*. and is not relevant to the choice of reflections for refinement. *R*-factors based on *F*^2^ are statistically about twice as large as those based on *F*, and *R*- factors based on ALL data will be even larger.

### Fractional atomic coordinates and isotropic or equivalent isotropic displacement parameters (Å^2^)

**Table:**

	*x*	*y*	*z*	*U*_iso_*/*U*_eq_	
Br1	0.65084 (6)	0.55540 (4)	0.27810 (3)	0.06465 (19)	
Br2	0.91872 (7)	0.04342 (5)	0.24015 (3)	0.07118 (19)	
C1	0.8141 (5)	0.4593 (4)	−0.1539 (3)	0.0496 (9)	
C2	0.8305 (6)	0.3098 (4)	−0.1488 (3)	0.0595 (11)	
H2A	0.9522	0.2786	−0.1815	0.071*	
H2B	0.7384	0.2722	−0.1794	0.071*	
C3	0.8005 (6)	0.2664 (4)	−0.0430 (3)	0.0574 (10)	
H3A	0.6953	0.2096	−0.0262	0.069*	
H3B	0.9093	0.2181	−0.0279	0.069*	
C4	0.7646 (5)	0.3963 (3)	0.0089 (3)	0.0428 (8)	
C5	0.7302 (5)	0.4116 (4)	0.1088 (3)	0.0507 (9)	
H5A	0.7291	0.3373	0.1505	0.061*	
C6	0.6988 (5)	0.5365 (4)	0.1428 (3)	0.0454 (9)	
C7	0.7025 (5)	0.6556 (3)	0.0827 (2)	0.0406 (8)	
C8	0.6704 (5)	0.7859 (3)	0.1171 (3)	0.0480 (9)	
H8A	0.6451	0.7985	0.1830	0.058*	
C9	0.6756 (5)	0.8937 (4)	0.0558 (3)	0.0540 (10)	
H9A	0.6544	0.9794	0.0801	0.065*	
C10	0.7121 (5)	0.8774 (4)	−0.0427 (3)	0.0527 (9)	
H10A	0.7146	0.9523	−0.0840	0.063*	
C11	0.7444 (5)	0.7531 (3)	−0.0796 (3)	0.0477 (9)	
H11A	0.7701	0.7435	−0.1459	0.057*	
C12	0.7392 (5)	0.6376 (3)	−0.0175 (2)	0.0397 (8)	
C13	0.7711 (5)	0.5055 (4)	−0.0526 (3)	0.0416 (8)	
C14	0.6249 (5)	−0.0807 (4)	0.6648 (3)	0.0528 (10)	
C15	0.5904 (6)	−0.2280 (4)	0.6573 (3)	0.0656 (12)	
H15A	0.6664	−0.2829	0.6938	0.079*	
H15B	0.4615	−0.2452	0.6825	0.079*	
C16	0.6392 (5)	−0.2604 (4)	0.5528 (3)	0.0551 (10)	
H16A	0.5316	−0.2878	0.5296	0.066*	
H16B	0.7347	−0.3316	0.5420	0.066*	
C17	0.7091 (4)	−0.1300 (3)	0.5032 (3)	0.0435 (9)	
C18	0.7752 (5)	−0.1078 (4)	0.4049 (3)	0.0498 (9)	
H18A	0.7815	−0.1767	0.3620	0.060*	
C19	0.8295 (5)	0.0155 (4)	0.3739 (3)	0.0462 (9)	
C20	0.8236 (4)	0.1243 (3)	0.4364 (3)	0.0416 (8)	
C21	0.8801 (5)	0.2544 (4)	0.4031 (3)	0.0491 (9)	
H21A	0.9247	0.2718	0.3381	0.059*	
C22	0.8680 (5)	0.3541 (4)	0.4681 (3)	0.0569 (11)	
H22A	0.9033	0.4396	0.4459	0.068*	
C23	0.8054 (5)	0.3321 (4)	0.5651 (3)	0.0543 (10)	
H23A	0.8014	0.4011	0.6077	0.065*	
C24	0.7489 (5)	0.2069 (4)	0.5980 (3)	0.0542 (10)	
H24A	0.7043	0.1923	0.6633	0.065*	
C25	0.7569 (5)	0.1006 (3)	0.5352 (3)	0.0429 (9)	
C26	0.7001 (5)	−0.0297 (3)	0.5667 (3)	0.0414 (8)	
O1	0.8324 (5)	0.5286 (3)	−0.2259 (2)	0.0722 (9)	
O2	0.5922 (4)	−0.0199 (3)	0.7378 (2)	0.0781 (9)	

### Atomic displacement parameters (Å^2^)

**Table:**

	*U*^11^	*U*^22^	*U*^33^	*U*^12^	*U*^13^	*U*^23^
Br1	0.0910 (4)	0.0609 (3)	0.0390 (3)	−0.0119 (2)	−0.0041 (2)	0.00645 (18)
Br2	0.0765 (3)	0.0813 (4)	0.0490 (3)	−0.0153 (2)	0.0092 (2)	−0.0078 (2)
C1	0.045 (2)	0.051 (2)	0.054 (2)	0.0024 (17)	−0.0130 (19)	−0.0079 (19)
C2	0.065 (3)	0.057 (3)	0.056 (3)	−0.003 (2)	−0.008 (2)	−0.015 (2)
C3	0.058 (3)	0.041 (2)	0.074 (3)	−0.0061 (18)	−0.011 (2)	−0.0051 (19)
C4	0.0401 (19)	0.037 (2)	0.051 (2)	−0.0083 (15)	−0.0050 (16)	−0.0017 (16)
C5	0.059 (2)	0.042 (2)	0.050 (2)	−0.0074 (18)	−0.0108 (19)	0.0109 (17)
C6	0.050 (2)	0.047 (2)	0.039 (2)	−0.0091 (17)	−0.0047 (17)	0.0022 (16)
C7	0.0395 (19)	0.041 (2)	0.041 (2)	−0.0082 (15)	−0.0069 (16)	0.0048 (15)
C8	0.053 (2)	0.045 (2)	0.045 (2)	−0.0002 (17)	−0.0088 (18)	−0.0033 (17)
C9	0.064 (3)	0.035 (2)	0.063 (3)	−0.0005 (18)	−0.013 (2)	−0.0006 (17)
C10	0.062 (3)	0.043 (2)	0.051 (2)	−0.0005 (18)	−0.0076 (19)	0.0106 (17)
C11	0.058 (2)	0.045 (2)	0.039 (2)	−0.0037 (18)	−0.0066 (18)	0.0081 (16)
C12	0.0379 (19)	0.0370 (19)	0.044 (2)	−0.0035 (15)	−0.0072 (16)	0.0030 (15)
C13	0.039 (2)	0.042 (2)	0.044 (2)	−0.0062 (15)	−0.0080 (16)	−0.0005 (15)
C14	0.045 (2)	0.061 (3)	0.054 (3)	−0.0040 (19)	−0.0141 (19)	0.012 (2)
C15	0.059 (3)	0.061 (3)	0.080 (3)	−0.013 (2)	−0.024 (2)	0.031 (2)
C16	0.046 (2)	0.038 (2)	0.083 (3)	−0.0077 (17)	−0.017 (2)	0.0108 (19)
C17	0.0309 (18)	0.042 (2)	0.059 (2)	−0.0021 (15)	−0.0142 (17)	0.0055 (17)
C18	0.045 (2)	0.041 (2)	0.064 (3)	−0.0022 (17)	−0.0090 (19)	−0.0090 (18)
C19	0.040 (2)	0.055 (2)	0.040 (2)	−0.0015 (17)	−0.0010 (16)	0.0007 (16)
C20	0.0318 (18)	0.041 (2)	0.051 (2)	−0.0002 (15)	−0.0073 (16)	0.0004 (16)
C21	0.043 (2)	0.048 (2)	0.058 (2)	−0.0121 (17)	−0.0121 (18)	0.0083 (18)
C22	0.059 (3)	0.034 (2)	0.081 (3)	−0.0123 (18)	−0.020 (2)	0.004 (2)
C23	0.053 (2)	0.045 (2)	0.067 (3)	−0.0079 (18)	−0.015 (2)	−0.016 (2)
C24	0.056 (2)	0.057 (3)	0.052 (2)	−0.0019 (19)	−0.013 (2)	−0.0053 (19)
C25	0.0360 (19)	0.042 (2)	0.052 (2)	0.0007 (15)	−0.0123 (17)	−0.0037 (16)
C26	0.040 (2)	0.042 (2)	0.044 (2)	−0.0029 (16)	−0.0117 (16)	0.0053 (15)
O1	0.098 (2)	0.079 (2)	0.0383 (17)	0.0139 (17)	−0.0135 (16)	−0.0042 (15)
O2	0.092 (2)	0.089 (2)	0.052 (2)	−0.0133 (18)	−0.0097 (17)	0.0082 (17)

### Geometric parameters (Å, °)

**Table:**

Br1—C6	1.898 (4)	C12—C13	1.421 (5)
Br2—C19	1.899 (4)	C14—O2	1.198 (5)
C1—O1	1.200 (5)	C14—C26	1.476 (5)
C1—C2	1.492 (5)	C14—C15	1.517 (6)
C1—C13	1.496 (5)	C15—C16	1.501 (6)
C2—C3	1.522 (6)	C15—H15A	0.9700
C2—H2A	0.9700	C15—H15B	0.9700
C2—H2B	0.9700	C16—C17	1.521 (5)
C3—C4	1.504 (5)	C16—H16A	0.9700
C3—H3A	0.9700	C16—H16B	0.9700
C3—H3B	0.9700	C17—C26	1.364 (5)
C4—C13	1.366 (5)	C17—C18	1.398 (5)
C4—C5	1.403 (5)	C18—C19	1.345 (5)
C5—C6	1.349 (5)	C18—H18A	0.9300
C5—H5A	0.9300	C19—C20	1.423 (5)
C6—C7	1.434 (5)	C20—C25	1.408 (5)
C7—C8	1.400 (5)	C20—C21	1.420 (5)
C7—C12	1.412 (5)	C21—C22	1.371 (5)
C8—C9	1.354 (5)	C21—H21A	0.9300
C8—H8A	0.9300	C22—C23	1.376 (6)
C9—C10	1.386 (5)	C22—H22A	0.9300
C9—H9A	0.9300	C23—C24	1.373 (6)
C10—C11	1.358 (5)	C23—H23A	0.9300
C10—H10A	0.9300	C24—C25	1.404 (5)
C11—C12	1.421 (5)	C24—H24A	0.9300
C11—H11A	0.9300	C25—C26	1.416 (5)
			
O1—C1—C2	126.2 (4)	O2—C14—C26	128.1 (4)
O1—C1—C13	126.9 (4)	O2—C14—C15	124.9 (4)
C2—C1—C13	106.9 (3)	C26—C14—C15	107.0 (3)
C1—C2—C3	107.6 (3)	C16—C15—C14	107.2 (3)
C1—C2—H2A	110.2	C16—C15—H15A	110.3
C3—C2—H2A	110.2	C14—C15—H15A	110.3
C1—C2—H2B	110.2	C16—C15—H15B	110.3
C3—C2—H2B	110.2	C14—C15—H15B	110.3
H2A—C2—H2B	108.5	H15A—C15—H15B	108.5
C4—C3—C2	103.8 (3)	C15—C16—C17	104.2 (3)
C4—C3—H3A	111.0	C15—C16—H16A	110.9
C2—C3—H3A	111.0	C17—C16—H16A	110.9
C4—C3—H3B	111.0	C15—C16—H16B	110.9
C2—C3—H3B	111.0	C17—C16—H16B	110.9
H3A—C3—H3B	109.0	H16A—C16—H16B	108.9
C13—C4—C5	120.9 (3)	C26—C17—C18	121.2 (3)
C13—C4—C3	112.5 (3)	C26—C17—C16	111.9 (4)
C5—C4—C3	126.6 (3)	C18—C17—C16	127.0 (3)
C6—C5—C4	118.4 (3)	C19—C18—C17	118.5 (3)
C6—C5—H5A	120.8	C19—C18—H18A	120.7
C4—C5—H5A	120.8	C17—C18—H18A	120.7
C5—C6—C7	123.8 (3)	C18—C19—C20	123.1 (4)
C5—C6—Br1	117.9 (3)	C18—C19—Br2	117.8 (3)
C7—C6—Br1	118.3 (3)	C20—C19—Br2	119.1 (3)
C8—C7—C12	119.0 (3)	C25—C20—C21	119.4 (3)
C8—C7—C6	124.4 (3)	C25—C20—C19	117.9 (3)
C12—C7—C6	116.7 (3)	C21—C20—C19	122.8 (4)
C9—C8—C7	121.0 (4)	C22—C21—C20	119.0 (4)
C9—C8—H8A	119.5	C22—C21—H21A	120.5
C7—C8—H8A	119.5	C20—C21—H21A	120.5
C8—C9—C10	120.6 (4)	C21—C22—C23	122.3 (4)
C8—C9—H9A	119.7	C21—C22—H22A	118.8
C10—C9—H9A	119.7	C23—C22—H22A	118.8
C11—C10—C9	120.6 (4)	C24—C23—C22	119.1 (4)
C11—C10—H10A	119.7	C24—C23—H23A	120.4
C9—C10—H10A	119.7	C22—C23—H23A	120.4
C10—C11—C12	120.4 (3)	C23—C24—C25	121.4 (4)
C10—C11—H11A	119.8	C23—C24—H24A	119.3
C12—C11—H11A	119.8	C25—C24—H24A	119.3
C7—C12—C13	119.1 (3)	C24—C25—C20	118.7 (4)
C7—C12—C11	118.4 (3)	C24—C25—C26	122.9 (4)
C13—C12—C11	122.5 (3)	C20—C25—C26	118.3 (3)
C4—C13—C12	121.1 (3)	C17—C26—C25	121.0 (3)
C4—C13—C1	109.1 (3)	C17—C26—C14	109.7 (3)
C12—C13—C1	129.7 (3)	C25—C26—C14	129.3 (3)
			
O1—C1—C2—C3	178.9 (4)	O1—C1—C2—C3	178.9 (4)
C13—C1—C2—C3	−1.1 (4)	C13—C1—C2—C3	−1.1 (4)
C1—C2—C3—C4	0.8 (4)	C1—C2—C3—C4	0.8 (4)
C2—C3—C4—C13	−0.2 (4)	C2—C3—C4—C13	−0.2 (4)
C2—C3—C4—C5	−179.3 (4)	C2—C3—C4—C5	−179.3 (4)
C13—C4—C5—C6	1.7 (5)	C13—C4—C5—C6	1.7 (5)
C3—C4—C5—C6	−179.3 (3)	C3—C4—C5—C6	−179.3 (3)
C4—C5—C6—C7	−1.4 (6)	C4—C5—C6—C7	−1.4 (6)
C4—C5—C6—Br1	179.6 (3)	C4—C5—C6—Br1	179.6 (3)
C5—C6—C7—C8	−179.9 (3)	C5—C6—C7—C8	−179.9 (3)
Br1—C6—C7—C8	−0.9 (5)	Br1—C6—C7—C8	−0.9 (5)
C5—C6—C7—C12	0.8 (5)	C5—C6—C7—C12	0.8 (5)
Br1—C6—C7—C12	179.8 (2)	Br1—C6—C7—C12	179.8 (2)
C12—C7—C8—C9	−0.5 (5)	C12—C7—C8—C9	−0.5 (5)
C6—C7—C8—C9	−179.8 (4)	C6—C7—C8—C9	−179.8 (4)
C7—C8—C9—C10	0.3 (6)	C7—C8—C9—C10	0.3 (6)
C8—C9—C10—C11	−0.4 (6)	C8—C9—C10—C11	−0.4 (6)
C9—C10—C11—C12	0.7 (6)	C9—C10—C11—C12	0.7 (6)
C8—C7—C12—C13	−179.7 (3)	C8—C7—C12—C13	−179.7 (3)
C6—C7—C12—C13	−0.5 (5)	C6—C7—C12—C13	−0.5 (5)
C8—C7—C12—C11	0.8 (5)	C8—C7—C12—C11	0.8 (5)
C6—C7—C12—C11	−179.9 (3)	C6—C7—C12—C11	−179.9 (3)
C10—C11—C12—C7	−0.9 (5)	C10—C11—C12—C7	−0.9 (5)
C10—C11—C12—C13	179.7 (3)	C10—C11—C12—C13	179.7 (3)
C5—C4—C13—C12	−1.4 (5)	C5—C4—C13—C12	−1.4 (5)
C3—C4—C13—C12	179.4 (3)	C3—C4—C13—C12	179.4 (3)
C5—C4—C13—C1	178.7 (3)	C5—C4—C13—C1	178.7 (3)
C3—C4—C13—C1	−0.5 (4)	C3—C4—C13—C1	−0.5 (4)
C7—C12—C13—C4	0.8 (5)	C7—C12—C13—C4	0.8 (5)
C11—C12—C13—C4	−179.8 (3)	C11—C12—C13—C4	−179.8 (3)
C7—C12—C13—C1	−179.3 (3)	C7—C12—C13—C1	−179.3 (3)
C11—C12—C13—C1	0.0 (6)	C11—C12—C13—C1	0.0 (6)
O1—C1—C13—C4	−179.0 (4)	O1—C1—C13—C4	−179.0 (4)
C2—C1—C13—C4	1.0 (4)	C2—C1—C13—C4	1.0 (4)
O1—C1—C13—C12	1.1 (7)	O1—C1—C13—C12	1.1 (7)
C2—C1—C13—C12	−178.9 (3)	C2—C1—C13—C12	−178.9 (3)
O2—C14—C15—C16	176.4 (4)	O2—C14—C15—C16	176.4 (4)
C26—C14—C15—C16	−2.3 (4)	C26—C14—C15—C16	−2.3 (4)
C14—C15—C16—C17	2.3 (4)	C14—C15—C16—C17	2.3 (4)
C15—C16—C17—C26	−1.6 (4)	C15—C16—C17—C26	−1.6 (4)
C15—C16—C17—C18	178.8 (3)	C15—C16—C17—C18	178.8 (3)
C26—C17—C18—C19	−0.3 (5)	C26—C17—C18—C19	−0.3 (5)
C16—C17—C18—C19	179.3 (3)	C16—C17—C18—C19	179.3 (3)
C17—C18—C19—C20	0.3 (5)	C17—C18—C19—C20	0.3 (5)
C17—C18—C19—Br2	−180.0 (2)	C17—C18—C19—Br2	−180.0 (2)
C18—C19—C20—C25	−0.2 (5)	C18—C19—C20—C25	−0.2 (5)
Br2—C19—C20—C25	−180.0 (2)	Br2—C19—C20—C25	−180.0 (2)
C18—C19—C20—C21	−179.6 (3)	C18—C19—C20—C21	−179.6 (3)
Br2—C19—C20—C21	0.6 (4)	Br2—C19—C20—C21	0.6 (4)
C25—C20—C21—C22	−0.1 (5)	C25—C20—C21—C22	−0.1 (5)
C19—C20—C21—C22	179.3 (3)	C19—C20—C21—C22	179.3 (3)
C20—C21—C22—C23	1.0 (5)	C20—C21—C22—C23	1.0 (5)
C21—C22—C23—C24	−1.5 (6)	C21—C22—C23—C24	−1.5 (6)
C22—C23—C24—C25	1.2 (6)	C22—C23—C24—C25	1.2 (6)
C23—C24—C25—C20	−0.4 (5)	C23—C24—C25—C20	−0.4 (5)
C23—C24—C25—C26	179.9 (3)	C23—C24—C25—C26	179.9 (3)
C21—C20—C25—C24	−0.2 (5)	C21—C20—C25—C24	−0.2 (5)
C19—C20—C25—C24	−179.6 (3)	C19—C20—C25—C24	−179.6 (3)
C21—C20—C25—C26	179.5 (3)	C21—C20—C25—C26	179.5 (3)
C19—C20—C25—C26	0.1 (5)	C19—C20—C25—C26	0.1 (5)
C18—C17—C26—C25	0.2 (5)	C18—C17—C26—C25	0.2 (5)
C16—C17—C26—C25	−179.4 (3)	C16—C17—C26—C25	−179.4 (3)
C18—C17—C26—C14	179.8 (3)	C18—C17—C26—C14	179.8 (3)
C16—C17—C26—C14	0.2 (4)	C16—C17—C26—C14	0.2 (4)
C24—C25—C26—C17	179.6 (3)	C24—C25—C26—C17	179.6 (3)
C20—C25—C26—C17	−0.1 (5)	C20—C25—C26—C17	−0.1 (5)
C24—C25—C26—C14	0.0 (6)	C24—C25—C26—C14	0.0 (6)
C20—C25—C26—C14	−179.7 (3)	C20—C25—C26—C14	−179.7 (3)
O2—C14—C26—C17	−177.3 (4)	O2—C14—C26—C17	−177.3 (4)
C15—C14—C26—C17	1.3 (4)	C15—C14—C26—C17	1.3 (4)
O2—C14—C26—C25	2.2 (6)	O2—C14—C26—C25	2.2 (6)
C15—C14—C26—C25	−179.1 (3)	C15—C14—C26—C25	−179.1 (3)

### Hydrogen-bond geometry (Å, °)

**Table:**

*D*—H···*A*	*D*—H	H···*A*	*D*···*A*	*D*—H···*A*
C15—H15A···O1^i^	0.97	2.54	3.495 (5)	167

Symmetry codes: (i) *x*, *y*−1, *z*+1.
